# Gut morphology and blood biochemical parameters of growing pigs fed diets with a partial to total replacement of soybean meal with *Arthrospira platensis*

**DOI:** 10.3389/fvets.2026.1728650

**Published:** 2026-02-24

**Authors:** Diana Giannuzzi, Alessandro Toscano, Gregorio Don, Rina Verdiglione, Stefano Schiavon, Luigi Gallo

**Affiliations:** Department of Agronomy, Food, Natural Resources, Animals and Environment (DAFNAE), University of Padova, Padua, Italy

**Keywords:** blood metabolites, fattening pigs, health, intestine, microalgae, nutrition

## Abstract

Soybean meal is the primary protein source in pig diets, but its production has been associated with deforestation, soil degradation, and loss of biodiversity. *Arthrospira platensis* (AP), a protein-rich cyanobacterium with a favorable amino acid profile, might represent a sustainable alternative. This study aimed to assess the impact of partial to full replacement of soybean meal with AP on gut morphology and blood biochemical parameters in growing-finishing pigs. A total of 88 barrows and gilts were assigned to one of the four isoenergetic, isoproteic, and isoaminoacidic diets: a control diet (100% soybean meal as a protein source) and experimental diets in which 33, 66%, or 100% of soybean meal were replaced with AP. Individual blood samples were collected at the start of the trial (91 days), and prior to slaughter (238 days), when pigs’ body weight averaged 41 ± 3 kg and 175 ± 6 kg, respectively. Ileum and colon tissues were sampled at the slaughterhouse for histological analysis and evaluation of gut morphology. Across dietary treatments, biochemical profiling indicated metabolic, oxidative, and inflammatory stability, with no alterations in liver or kidney-related parameters. Consistently, histological evaluation indicated that intestinal architecture was preserved across all the dietary treatments, even though ileal villus width and crypt depth tended to increase linearly with higher dietary inclusion of AP, and a similar trend was observed for ileal inflammation score. Despite reports in other species suggesting AP antioxidative and anti-inflammatory benefits, such effects were not evident in pigs under the conditions tested. Nonetheless, the absence of adverse effects on intestinal and systemic health supports the nutritional viability of AP as a complete replacement for soybean meal. These findings highlight its potential as a sustainable and safe protein source in swine production without compromising physiological status or gut health.

## Introduction

In livestock systems, protein is the most expensive and limiting nutrient in diet formulations (1). The production and supply of protein feeds are crucial due to their significant environmental impacts, including land use change, land occupation, and the consumption of energy, water, and fertilizers (2). Furthermore, the intensification of the feed–food competition and the consequent potential negative effects on feed security should also be taken into consideration (3). Focusing on the European pig sector, soybean meal is the first choice among protein feeds due to both its nutritional quality and accessibility (4). Moreover, product specifications for pig-derived typical products, such as those related to Protected Designation of Origin Italian dry-cured hams, support the use of soybean meal as the primary protein source in the diets of growing and finishing pigs (5).

Therefore, alternatives to land-based soybean meal production will be necessary to ensure the future viability of animal productions (6). Using the soybean meal as a reference, potential alternative protein sources should provide a high amount of protein of suitable biological value, should allow for the efficient use of land and water, and should not impair product quality and animal health status (7). Aquatic biomass production could be an interesting alternative, as these microorganisms grow well with minimal land use (8). Among these biomass, *Arthrospira platensis* (AP) has gained particular interest. It is a prokaryotic, multicellular cyanobacterium, commonly known as the microalga *Spirulina* due to its morphology and photosynthetic activity. Its nutritive characteristics include a high crude protein content (over 60% dry matter, DM), which is much greater than that of the soybean meal (approximately 45%), and a well-balanced amino acid profile (8, 9). Studies dealing with the use of AP in pig nutrition are still scarce and have primarily been conducted using minimal dietary inclusion levels as a supplement. Few studies have included AP as a feed ingredient in the nutrition of growing pigs, reporting controversial effects on growth parameters and no adverse effects on carcass characteristics (10, 11).

Apart from evaluating productive performance and product quality, some studies have investigated the effects of AP administration at both local and systemic levels. In the case of monogastric species, the impact of this cyanobacterium on gut health has garnered significant attention because of its potential to enhance it (12). In this context, variations of histomorphometric parameters of the intestinal tract are critical for evaluating nutrient absorption and gut health (13). Aquatic biomass-derived oligosaccharides and polysaccharides may act as selective substrates for beneficial gut bacteria, particularly within the ileal tract (14, 15). AP supplementation in chickens has been associated with an increased villus height and an improved villus-to-crypt ratio. This contributes to a greater intestinal surface area for nutrient absorption, which in turn may enhance digestive enzyme activity and overall feed efficiency (16). Moreover, in broilers, Ansari et al. (17) have reported a dose-dependent effect in the dietary inclusion of AP, yielding the most pronounced improvements in histomorphometric indices across all major intestinal sections. In weaning and post-weaning piglets, even at low inclusion levels, AP supplementation has shown immunomodulatory effects and a reduction in diarrhea incidence (18–20). However, results regarding its impact on intestinal histomorphology remain inconsistent (19). Hence, no studies have thoroughly investigated the effects of AP on intestinal morphology of growing and finishing animals, particularly under conditions relevant to the total replacement of soybean meal with this novel protein source.

In addition, non-morphological mechanisms have been suggested to exert beneficial effects on gut health via antioxidant and immune system modulation induced by AP-derived components transferred to the liver (21, 22). In this regard, biochemical blood parameters are widely regarded in evaluating animals’ health and nutritional status (23). The process of collecting these parameters is relatively non-invasive and encompasses a wide range of physiological processes occurring simultaneously in the organism (24, 25). Specifically, blood biochemistry is frequently used at the herd level, serving as an initial warning system for identifying subclinical dysfunctions. Few studies have been conducted on the effects of microalgae on the blood profile of livestock animals, and the existing research has largely included them at low dietary levels as supplements. A daily AP administration of 0.5–1.0 g/10 kg BW reduced blood glucose, cholesterol, serum malondialdehyde, and liver aminotransferase in small ruminants (26, 27). In a recent study, Spinola et al. (22) reported that the effects of AP administration, mostly as a dietary supplement, on blood health markers of broilers are complex and related to its level of inclusion. Fattening pigs supplemented with *Spirulina maxima* enriched with Cu indicated a reduction in low-density lipoprotein and total cholesterol, along with changes in liver enzyme activities, suggesting an improved hepatic metabolic profile (28). In contrast, the study of Martins et al. (20) reported increased concentrations of plasma cholesterol, total lipids, ALT, AST, and alkaline phosphatase in weaned pigs receiving a diet with 10% AP. Therefore, a comprehensive analysis of blood metabolic parameters under conditions of high AP levels used as a replacement for traditional protein source feeds in growing pigs and until they reached heavy market weights is lacking and needs to be assessed to effectively determine the consequences of using this ingredient in pig feeding, beyond productive performance.

To fill these gaps, in this study, planned within a more comprehensive feeding trial aimed at evaluating the effects of using AP as the primary dietary ingredient on growth and carcass traits of growing-finishing pigs (29), we performed gut morphology evaluations and a complete blood metabolic profile, including 24 metabolites, on 88 pigs reared from early growing to finishing stages with progressive to total replacement (33, 66, and 100%, respectively) of soybean meal with AP. The aim was to investigate the effects of different inclusion levels of AP on intestinal morphology and blood biochemical parameters to comprehensively assess its potential local and systemic impacts.

## Materials and methods

### Animals, diets, and experimental procedures

Experimental details of the feeding trial in which the present research has been carried out are reported in Don et al. (29). Briefly, this study involved 88 Goland-C21 × Camborough-43 pigs, 37 gilts, and 51 barrows, born on the same day at a commercial sow farm. At 82 days of age (average body weight—BW 41.3 ± 3.4 kg), pigs were moved to the experimental pig unit of the Department of Agronomy, Food, Natural Resources, Animals and Environment (DAFNAE) Department of the University of Padova and allotted to 8 pens (11 pigs/pen) balanced for BW and sex. After an acclimation period of 2 weeks, during which all animals were fed the same diet, the feeding regimen was modified to include four different diets (two pens per diet): a commercial feeding regimen, traditionally used for the production of heavy pigs aimed to provide typical dry-cured hams and based on cereals and soybean meal, set as control, and three diets in which soybean meal was progressively substituted with increasing percentage of a nucleus containing a cultivated spray-dried AP powder (Aim Grow Biotech Co., Ltd., Port Coquitlam, BC, Canada), specifically 33, 66, and 100% (AP33, AP66 and AP100, respectively). The AP nucleus was formulated to mimic the nutrient contents of soybean meal, which had a lower CP content than the AP used in this study (30), and allowed to obtain isoenergetic, isoproteic, and isoaminoacidic dietary treatments. The ingredient and chemical compositions of the diets fed at the start of the trial, prior to the soybean meal replacement (acclimation period), and at the end of the trial (finishing period) are reported in Table 1.

**Table 1:** Ingredients and main nutrients composition of the experimental diets at the start of the trial, before *A. platensis* administration (T0), and at the end of the trial (T1).

Item	Diet at T0	Diet^a^ at T1			
	Acclimation feed	CTR	AP33	AP66	AP100
Ingredient (g/kg DM)					
Corn grain	582.3	586.3	585.8	583.9	582.7
Barley grain	176.2	249.8	249.6	248.8	248.3
Wheat middlings	66.6	56.1	55.9	55.8	55.6
Wheat bran	32.7	32.9	32.8	32.7	32.7
Lard	11.4	11.4	11.4	11.4	11.4
SP nucleus^b^	0.0	0.0	45.3	90.3	135.1
Soybean meal	203.6	133.0	88.5	44.2	0.0
Calcium carbonate	17.7	17.8	17.7	17.6	17.6
Silica^c^	-	12.4	12.3	12.3	12.3
Sodium chloride	4.9	4.9	4.9	4.9	4.9
Dicalcium phosphate	4.7	4.8	4.8	4.8	4.8
Vitamin mineral premix^d^	1.9	1.9	1.9	1.9	1.9
L-Lysine monohydrocloride^e^	0.8	0.0	0.0	0.0	0.0
OptiPhos (phytase)^f^	1.2	1.2	1.2	1.2	1.2
Nutrient composition (g/kg DM)					
Dry matter (DM)	906	899	899	901	902
Crude protein	170	145	146	145	146
Ether extract	40	42	43	44	43
NDF	136	158	152	154	153
Starch	555	449	449	450	448
Ash	54	48	56	50	51
Lysine	6.8	7.3	7.2	7.4	7.6
Methionine	1.3	2.4	2.8	2.7	3.1
Threonine	4.5	4.8	5.0	5.1	5.5
Tryptophan	1.4	1.3	1.2	1.5	1.4
Tyrosine	2.9	2.6	2.7	2.7	2.9
Ca (g/kg)	11.0	9.6	11.6	11.0	10.1
P (g/kg)	4.4	4.8	5.0	5.1	5.1
Na (g/kg)	0.9	2.0	2.1	2.2	2.2

a CTR = control diet; AP33 = diet with substitution of soybean with 33% of *A. platensis* nucleus; AP66 = diet with substitution of soybean with 66% of *A. platensis* nucleus; AP100 = diet with total substitution of soybean with *A. platensis* nucleus.

b Ingredient composition: *Arthrospira platensis* 640 g/kg; sugar beet pulp 308 g/kg; soft wheat 40 g/kg; L-Lysine Monoclohydrate 6 g/kg; L-triptophan 6 g/kg.

c Silica (silica granular 10.SiO_2_.H_2_O, Impextraco, Heist-op-den-Berg, Belgium) was included to increase the acid-insoluble ash content as a marker for a digestibility study carried out within the feeding trial.

d Providing per kilogram of feed: vitamin A, 8000 IU; vitamin D3, 1,200 IU; vitamin E, 8 mg; vitamin B7, 0.08 mg; vitamin B12, 0.012 mg; niacin, 16.0 mg; biotin, 8 mg; iron, 170 mg; zinc, 117 mg; copper, 14 mg; cobalt, 0.11 mg; iodine, 0.06 mg; manganese, 65 mg; magnesium, 0.14 mg; selenium 10 mg.

e L-Lysine Monohydrochloride, 98.5% pure, 78% L-Lysine (Methodo Chemicals, 42,017 Novellara, RE, Italy).

f Optiphos^®^ (Phytase, Huvepharma).

Chemical composition (g/kg): Dry matter 913; Crude protein (N × 6.25) 467; Lysine 28; Methionine+cysteine 13.2; Threonine 21.5; Triptophan 6.9; Starch 25; Ether extract 47; Ash 64.

All pens were equipped with a single-space electronic feeder (Compident MLP 2—SMARTCON, Schauer Agrotronic, Prambachkirchen, Austria), which allowed to individually feed pigs according to a mild restricted feeding curve adjusted every 2 weeks, providing 1.90 to 3.20 kg/d from the first to the last week on feed. Pens were also equipped with nipple drinkers to allow free drinking to animals.

### Serum metabolic profile

Blood samples were collected from the jugular vein of each pig using 9 mL vacuum tubes (FL Medical s.r.l., Torreglia, Padova, Italy) at two different times: during the acclimation period (91 days of age and 46.8 kg as mean BW, T0) and at the end of finishing period, before slaughter (238 days of age and 174.5 kg as mean BW, T1). All samples were refrigerated at 4 °C until transfer to the laboratory of the Experimental Zooprophylactic Institute of Venezie (IZSVe, Legnaro, Italy) for analysis. The following biochemical parameters were analyzed in serum using a Cobas Pure e303 analyzer (Roche Diagnostics, Mannheim, Germany): total proteins (PROTt), albumin (ALB), urea, creatinine, glucose, cholesterol (Chol), triglycerides (TG), total and direct bilirubin (BILt and BILd), aspartate transaminase (AST), alanine transaminase (ALT), alkaline phosphatase (ALP), *γ*-glutamyl transferase (GGT), creatinine kinase (CK), lactate transaminase (LDH), calcium (Ca), phosphorus (P), magnesium (Mg), sodium (Na), potassium (K), chlorine (Cl), and iron (Fe). The concentrations of reactive oxygen metabolites (ROM) and plasma protection against oxidation (hypochlorite-induced oxidation of protein in plasma, OXY) were measured using a colorimetric method (Diacron Labs, Grosseto, Italy) applied to the Cobas Pure e303 analyzer. A different colorimetric method (Tridelta Development Ltd., Co. Kildare, Irlanda) was used to determine haptoglobin (Hp) concentration. Globulin concentration was calculated by the difference between albumin and total protein. Reference ranges for all serum metabolites were provided by IZSVe (Legnaro, Italy), except for ROM and OXY, for which reference ranges are not defined; consequently, these parameters were evaluated using a tertile-based classification.

### Gut sampling and histological analysis

Gut tissue samples were collected at the slaughterhouse from each animal. For every pig, one ileal segment (60 cm proximal to the ileocaecal junction) and one segment from the proximal colon (20 cm aboral to the ileocecal valve) were excised, flushed with a 0.9% NaCl solution, and fixed into 10% neutral formalin.

Samples were embedded in paraffin, sectioned at a 7-μm thickness, and stained with hematoxylin–eosin. For each intestinal sample, between three and five histological sections were prepared. Stained samples were evaluated using an Axioscope 5 (Carl Zeiss, Jena, Germany), and images were captured with an Axiocam 208 color camera (Carl Zeiss, Jena, Germany) under 2.5x, 10x, 20x, and 40 × magnification. The measurements were performed using the ZEISS ZEN 3.11 software (Carl Zeiss, Jena, Germany). Sections prepared were used to select 4–5 well-oriented intact and complete villi and crypts from the mucosa of the ileum and 4–5 crypts of the proximal colon intestinal region for the histological measurements: villus height (VH) was measured from the tip to the base of the villus, villus width (VW) was measured at the base of the villus, and crypt depth (CD) was measured from tip of the crypt to the point where it meets the muscularis mucosa (31, 32). The villus-to-crypt ratio was determined as VH/CD. The mean value based on eight measurements was reported for each animal.

The inflammation status of the ileum and the colon was assessed in a blinded manner using a scoring system ranging from 1 to 4, adapted from the criteria described by Erben et al. (33), which integrated both the severity and the extent of inflammatory cell infiltration. Specifically, a score of 1 corresponded to minimal (<10%) leukocyte infiltration, limited to the mucosa. Score 2 indicated leukocyte mild infiltration (10–25%) in the mucosa and submucosa. Score 3 reflects moderate (26–50%) leukocyte infiltration involving both the mucosa and submucosa. Finally, score 4 indicates dense leukocyte infiltration in the mucosa and submucosa (>50%).

### Statistical analysis

Blood biochemical parameters were analyzed using two different models, both run using the PROC MIXED procedure implemented in SAS software (SAS Inst. Inc., Cary, NC).

To highlight the putative differences associated with the period of blood sampling and sex, the following linear mixed model was used:


$$\begin{array}{c} y_{\text{ijklmn}}=\mu +\text{perio}d_{i}+\mathrm{se}x_{j}+\mathrm{die}t_{k}+{\left(\text{period}\;\times \;\mathrm{sex}\right)}_{\mathrm{ij}}\\ +{\left(\text{period}\;\times \;\text{diet}\right)}_{\mathrm{ik}}+{\left(\mathrm{sex}\;\times \;\text{diet}\right)}_{\mathrm{jk}}+\mathrm{pen}{\left(\text{diet}\right)}_{k:l}\\ +\text{animal}{\left(\mathrm{sex}\right)}_{j:m}+e_{\text{ijklmn}} \end{array}$$

Where *y*_*ijklmn*_ was the observed trait; *μ* was the overall intercept of the model, *period* was the fixed effect of the *i*^th^ period of blood sampling (*i*: 1 = T0, 2 = T1); *sex*_*j*_ was the fixed effect of the *j*^th^ sex (*j*: 1 = gilts, 2 = barrows); *diet*_*k*_ was the fixed effect of the *k*^th^ diet (*j* = 1, …, 4); (*period* × *sex*)_*ij*_, (*period* × *diet*)_*ik*_, and (*sex* × *diet*)_*jk*_ were the interaction effects between *sex*_*j*_ and *period*_*i*_, *diet*_*k*_ and *period*_*i*_ and *sex*_*j*_ and *diet*_*k*_, respectively; *pen*_*l*_ was the random effect of the *l*^th^ pen (*l* = 1,…,8) within *diet*_*k*_; *animal*_*m*_ was the random effect of the *m*^th^ animal (m = 1,…,88) within *sex*_*j*_; and *e*_*ijklmn*_ was the random residual. The pen, animal, and residual effects were assumed to be independent and normally distributed with a mean of zero and variances σ^2^_l_, σ^2^_k_, and σ^2^_e_, respectively.

Considering that pigs allotted to the different experimental groups received the same diet during the first period of blood sampling (T0), blood biochemical parameters were analyzed separately at the two-sampling times (T0 and T1) to highlight the putative effects associated with the different diets, according to the following linear mixed model:


$$y_{\text{ijkl}}=\mu +\mathrm{se}x_{i}+\mathrm{die}t_{j}+{\left(\mathrm{sex}\times \text{diet}\right)}_{\mathrm{ij}}+\mathrm{pen}{\left(\text{diet}\right)}_{k:j}+e_{\text{ijkl}}$$

Where *y*_*ijkl*_ was the observed trait; *μ* was the overall intercept of the model; *sex*_*i*_ was the fixed effect of the *i*^th^ *sex* (*i*: 1 = gilts, 2 = barrows); *diet*_*j*_ was the fixed effect of the *j*^th^ experimental group (T0, *j* = 1, …, 4) or of the *j*^th^ diet (T1, *j* = 1, …, 4); (*sex* × *diet*)_*ij*_ was the interaction effect between *sex*_*i*_ and *diet*_*j*_; *pen*_*k*_ was the random effect of the *k*^th^ pen (l = 1,…,8) within *diet*_*j*_; and *e*_*ijkl*_ was the random residual. The pen and residual effects were assumed to be independently and normally distributed with a mean of zero and variances of σ^2^_l_, σ^2^_k_, and σ^2^_e_, respectively. Differences between the least square means of the different diets were adjusted using the Bonferroni correction method and considered significant at a *p*-value of ≤ 0.05. The same linear mixed model and the same criteria of least square means comparison were also applied to the histological measurements.

In addition, orthogonal polynomial contrasts were performed to test linear, quadratic, and cubic trends associated with increasing levels of AP in the diet. Differences were declared significant at a *p-*value of ≤ 0.05.

## Results

### Diet and performance traits

All pigs were fed the same diet without AP administration in the first two weeks of trial (T0, acclimation period) and were subsequently switched to diets differing in AP content. The AP nucleus, formulated to progressively replace soybean meal with AP, which included AP powder, sugar beet pulp, and wheat meal, closely matched the chemical composition of soybean meal and allowed for effective replacement of the conventional protein source without altering the dietary balance (Table 1).

Statistics concerning main performance traits are reported in Table 2. The animals started with an average body weight (BW) of 41.3 ± 3.4 kg and reached 174.9 ± 6.3 kg at the end of the trial. Their average daily gain was 888 g, supported by a feed intake of 2,622 g/d, resulting in a gain-to-feed ratio of 0.339 g/g.

**Table 2:** Descriptive statistics of the main performance traits of pigs (*n* = 86).

Trait	Mean	SD^a^	Minimum	Maximum
Initial body weight, kg	41.6	3.4	32.5	51.0
Final body weight, kg	174.9	6.37	157.5	187.0
Average daily gain, g/d	888	40	790	978
Feed intake, g/d	2,622	57	2,362	2,699
Gain:feed, g/g	0.339	0.015	0.304	0.378

a SD: standard deviation.

### Histological evaluation

**Figure 1:**
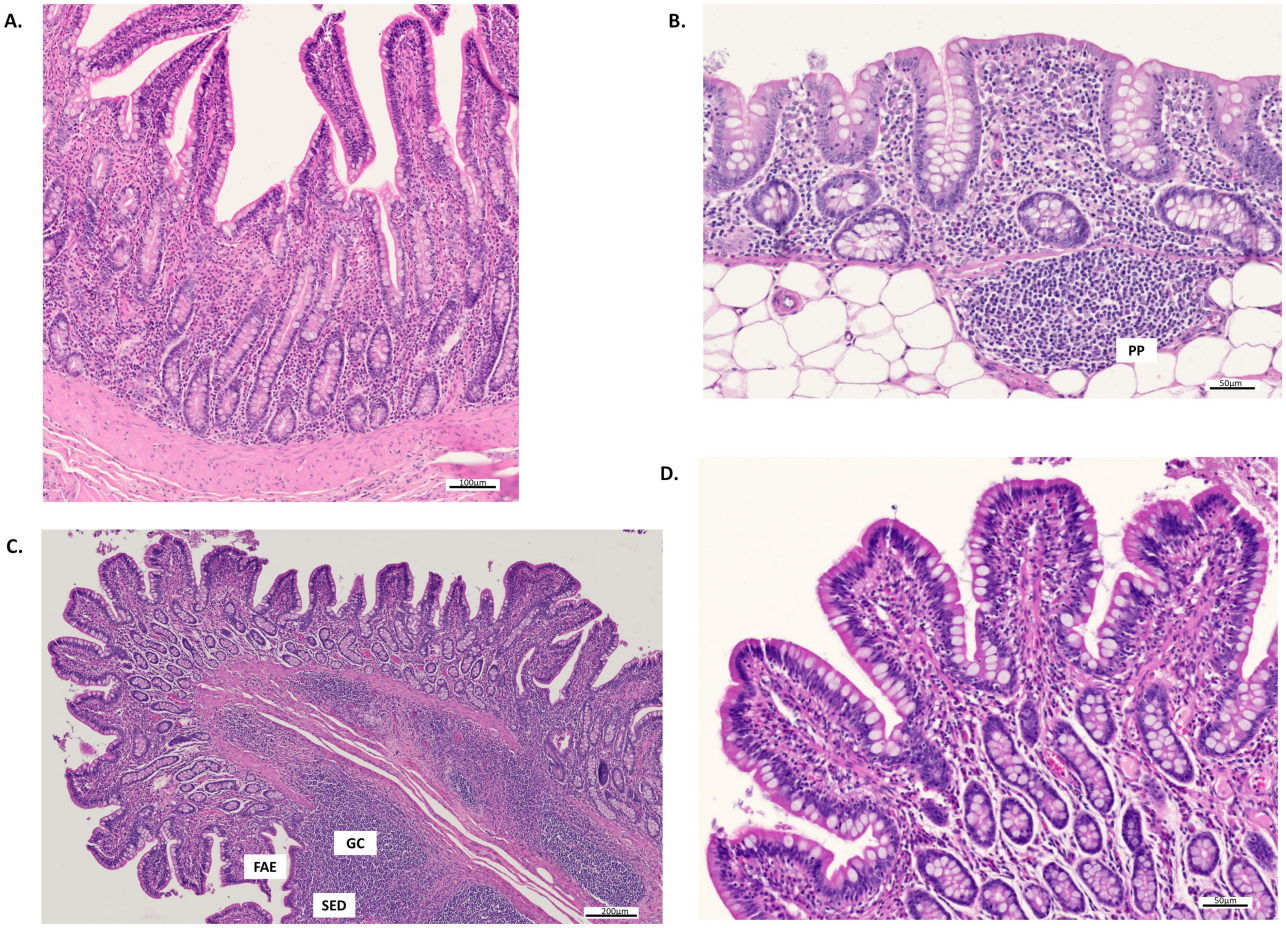
Histological micrographs of the colon and ileum stained with hematoxylin–eosin from pigs fed CTR and AP33 diets. **(A)** Ileum, a cross-section of mucosa and submucosa from pigs fed the control diet, showing scattered eosinophilic leukocytes in the mucosa and numerous goblet cells in the epithelium of villi and crypts. Scale bar: 100 μm. **(B)** Colon, a cross-section of mucosa and submucosa from pigs fed the control diet, where a Peyer’s patch (PP) is evident. Scale bar: 50 μm. **(C)** Ileum, a cross-section of mucosa and submucosa from pigs fed the AP33 diet. The germinal center (GC), subepithelial dome (SED), and follicle-associated epithelium (FAE) of the PPs are indicated. Scale bar: 200 μm. **(D)** Detail of panel C, highlighting scattered eosinophilic leukocytes in the mucosa and numerous goblet cells in the epithelium. Scale bar: 50 μm.

**Figure 2:**
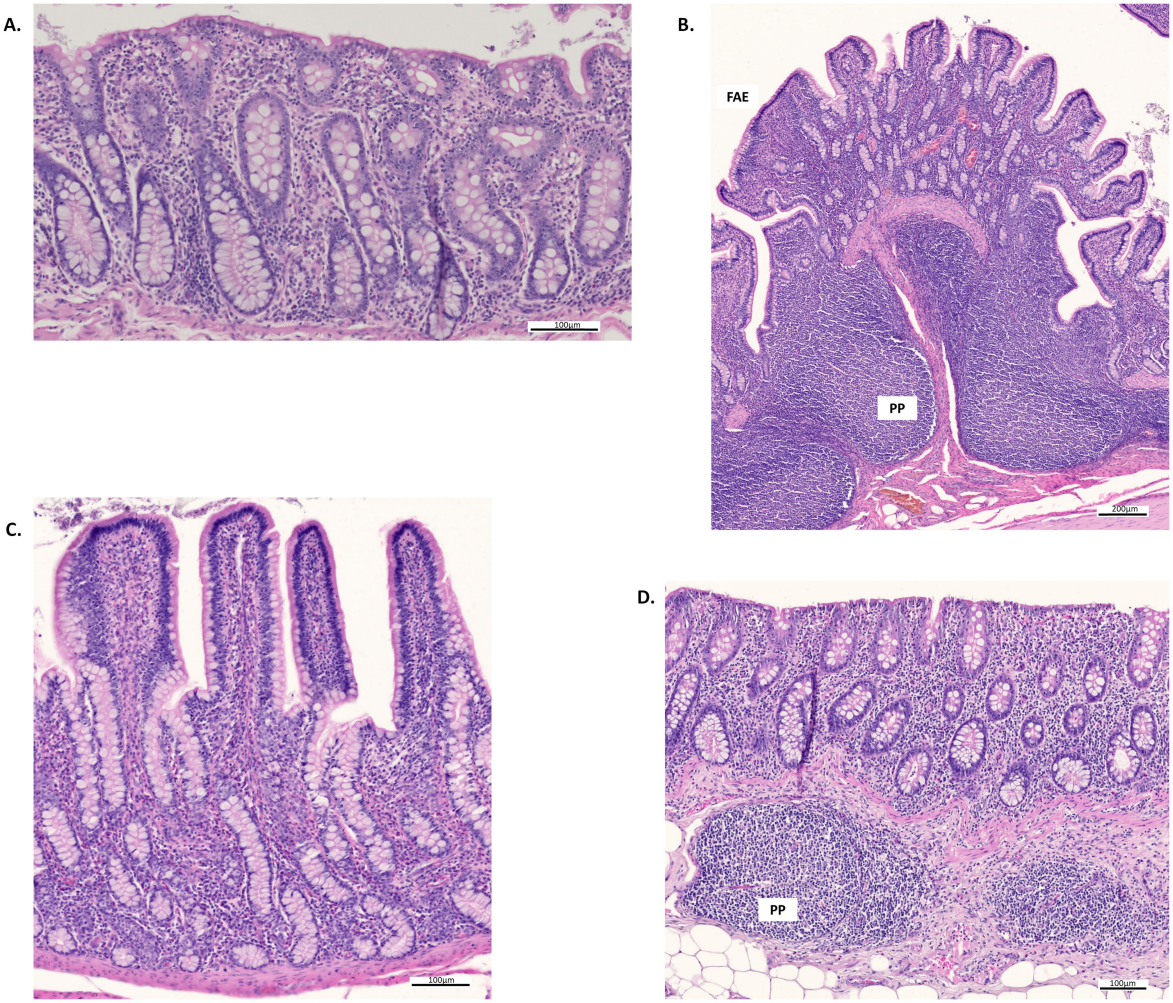
Histological micrographs of the colon and ileum stained with hematoxylin–eosin from pigs fed the AP66 and AP100 diets. **(A)** Colon, a cross-section of mucosa from pigs fed the AP66 diet, showing scattered eosinophilic leukocytes and an epithelium rich in goblet cells. Scale bar: 100 μm. **(B)** Ileum, a cross-section of mucosa and submucosa with Peyer’s patches (PPs) from pigs fed the AP100 diet. Numerous goblet cells are present in the epithelium, except within the follicle-associated epithelium (FAE). Scale bar: 200 μm. **(C)** Ileum, a cross-section of mucosa from pigs fed the AP100 diet, showing moderate leukocyte infiltration and abundant goblet cells located along the sides of the villi and within the crypts. Scale bar: 100 μm. **(D)** Colon, a cross-section of mucosa and submucosa with PPs from pigs fed the AP100 diet, displaying moderate leukocyte infiltration in the mucosa and numerous goblet cells in the crypts. Scale bar: 100 μm.

Histological micrographs of the ileum and colon sections, captured at various magnifications under the different dietary treatments, are presented in Figure 1 (CTR and AP33) and Figure 2 (AP66 and AP100). The results of an analysis of variance (ANOVA) assessing the effects of diet and sex on intestinal morphology traits and inflammation score are presented in Table 3. In general, diet effect did not significantly affect any of the histomorphological gut parameters evaluated. However, VW (*p* = 0.08) and CD (*p* = 0.07) in the ileum tended to increase linearly with the progressive substitution of soybean meal with AP.

**Table 3:** Least square means and results of ANOVA (F-values and level of significance) for diet and sex effects of intestinal morphology traits and inflammation score at the end of the trial (*n* = 86 pigs).

Item	Dietary treatments^a^				F-value^b^	Sex		F-value^b^	RMSE^c^
	CTR	AP33	AP66	AP100		Gilts	Barrows		
Villus height (VH, μm)									
Ileum	281	291	299	301	0.23	297	290	0.38	38.4
Villus width (μm)									
Ileum	129	133	134	142	1.15	137	132	1.12	18.9
Crypt depth (CD, μm)									
Ileum	304	305	333	333	1.36	308	329	2.13	51.1
Colon	360	350	346	348	0.22	358	344	0.99	51.7
VH/CD									
Ileum	0.95	0.97	0.91	0.93	0.29	1.00	0.89	5.60^*^	0.18
Inflammation score^d^									
Ileum	1.13	1.29	1.07	1.80	2.27	1.35	1.30	0.05	0.81
Colon	1.21	1.00	1.39	1.10	3.12	1.25	1.10	2.25	0.36

a CTR = control diet; AP33 = diet with substitution of soybean with 33% of *A. platensis* nucleus; AP66 = diet with substitution of soybean with 66% of *A. platensis* nucleus; AP100 = diet with total substitution of soybean with *A. platensis* nucleus.

b ^*^: *p* < 0.05.

c RMSE = root mean square error.

d Scores range from 1 (minimum inflammation) to 4 (maximum inflammation).

Moreover, without reaching statistical significance, pigs of the AP100 group evidenced a nominally more pronounced inflammatory response in the ileum than those of the control group (1.80 vs. 1.13), and the ileum inflammation score tended to increase linearly with the progressive substitution of soybean meal with AP (*p* = 0.07).

Furthermore, gilts and barrows did not differ significant in any histomorphological gut parameters, with the only exception of the ileum villus-to-crypt ratio, which appeared higher in gilts compared to barrows (*p* < 0.05).

### Blood biochemical parameters

The descriptive statistics of blood biochemical parameters grouped according to their physiological meaning are presented in Table 4 in order to provide typical mean values and variation for healthy pigs at young (T0, before AP administration) and very mature (T1, after AP administration) age and weight. In general, mature pigs at T1 evidenced slightly or mildly greater mean values for all of the blood parameters considered, with few exceptions, whereas a variation of parameters was comparable between T0 and T1.

**Table 4:** Descriptive statistics of the biochemical parameters at time 0 (T0: Start of the trial, before *A. platensis* administration, *n* = 88 pigs) and at time 1 (T1: end of the trial, *n* = 86 pigs).

Item^a^	T0					T1				
	Mean	SD^b^	CV^c^	P1	P99	Mean	SD	CV	P1	P99
Energy-related metabolites										
Glucose, mmol/L	5.68	0.72	12.6	3.9	7.4	5.83	1.2	20.5	2.2	9.6
Cholesterol, mmol/L	2.22	0.3	13.4	1.57	3.55	2.74	0.32	11.7	1.77	3.54
Creatinine, μmol/L	94.1	12.3	13.1	67	141	162	22	13.6	120	251
CK, U/L	1,812	2,288	126	190	11,440	1,969	145	7.35	1,057	2,000
TG, mmol/L	0.466	0.18	38.2	0.2	1.39	0.494	0.16	31.9	0.27	1.25
Urea, mmol/L	3.81	0.9	23.7	2	6.4	4.38	0.76	17.3	2.8	6.1
Liver function										
Albumin, g/L	40.1	3.08	7.69	32	46	50.5	2.96	5.85	39	57
ALP, U/L	208	40.3	19.4	97	314	134	61.7	46.2	51	536
ALT, U/L	49.6	7.55	15.2	32	71	62.9	9.17	14.6	36	81
AST, U/L	41.3	24	58.2	17	146	68.1	30.3	44.5	32	260
GGT, U/L	36	11.9	32.9	13	95	39.7	10.8	27.2	15	69
LDH, U/L	592	238	40.2	348	1,671	727	291	40	378	2,332
Inflammation										
Globulins, g/L	23.5	3.62	15.4	17	33	23.4	4.13	17.6	15	41
PROTt, g/L	63.6	3.47	5.45	54	72	73.9	3.42	4.63	68	87
Haptoglobin, mg/dL	89.1	46.5	52.2	20.5	285	128	92.6	72.4	20.6	646
Oxidative stress metabolites										
ROM, mgH_2_O_2_/100 mL	53.1	6.40	12.1	38.5	67.5	84.8	11.8	13.9	65.9	129
OXY, μmol HClO/mL	280	16.5	5.9	241	332	339	14	4.14	309	376
Minerals										
Ca, mmol/L	2.77	0.23	8.43	2.34	3.84	2.77	0.15	5.29	2.46	3.4
Cl, mmol/L	98.6	1.8	1.83	94	103	98.9	1.86	1.88	95	104
Fe, μg/dL	111	47.8	42.9	30	315	134	30.6	22.8	48	208
K, mmol/L	5.61	0.6	10.6	4.51	8.55	8.4	1.03	12.3	5.68	10.3
Mg, mmol/L	0.909	0.06	6.79	0.78	1.08	1.14	0.1	9.11	0.89	1.45
Na, mmol/L	143	1.82	1.27	138	147	146	3.37	2.31	138	153
P, mmol/L	2.94	0.41	13.9	1.94	4.17	3.3	0.28	8.53	2.51	3.92

a CK = creatin kinase; TG = triglycerides; ALP = alkaline phosphatase; ALT = alanine aminotransferase; AST = aspartate aminotransferase; GGT = γ-glutamyl transferase; LDH = lactate dehydrogenase; PROTt = total proteins; ROM = reactive oxygen metabolites; OXY = plasma protection against oxidation (hypochlorite-induced oxidation of protein in plasma).

b SD: standard deviation.

c CV: coefficient of variability.

Least square means of experimental groups at T0 and at T1 are presented in Table 5. Data from the two periods were analyzed separately because, at T0, pigs were fed the same diet, and the experimental group represented only a figurative effect, whereas, at T1, pigs were fed different diets for the duration of the trial, and the experimental groups expressed different dietary treatments. As expected, at T0, pigs allotted to different experimental groups did not differ for any of the blood biochemical parameters considered. Similarly, at T1, pigs allotted to different dietary treatments did not differ, and the diet effect never approached statistical significance. More specifically, the progressive replacement of soybean meal with AP did not influence the blood parameters considered, and no clear pattern of variation was observed with increasing AP inclusion.

**Table 5:** Least square means and results of ANOVA (F-values and level of significance) of biochemical parameters at the start of the trial, before *A. platensis* administration (T0), and at the end of the trial (T1), for pigs allotted to different experimental groups (T0) or fed different dietary treatments (T1).

Item^a^	T0						T1					
	Experimental groups^b^				F-value	RMSE^c^	Dietary treatments				F-value	RMSE
	CTR	AP33	AP66	AP100			CTR	AP33	AP66	AP100		
Energy-related metabolites												
Glucose, mmol/L	5.54	5.90	5.63	5.64	0.223	0.65	5.83	5.43	6.45	5.55	0.140	1.15
Total Cholesterol, mmol/L	2.29	2.16	2.15	2.20	0.383	0.26	2.64	2.75	2.81	2.80	0.347	0.296
Creatinine, μmol/L	92.7	94.2	93.8	98.4	1.357	11.5	156	162	161	167	0.636	21.9
CK, log	3.16	2.89	3.05	2.98	1.560	1	3.29	3.30	3.29	3.30	1.282	0.096
TG, log	−0.339	−0.407	−0.317	−0.378	1.437	0.307	−0.314	−0.278	−0.355	−0.327	0.706	0.268
Urea, mmol/L	3.97	3.71	3.82	3.57	0.308	0.8	4.38	4.53	4.26	4.27	0.178	0.742
Liver function												
Albumin, g/L	41.7	39.0	39.8	39.4	1.356	2.81	50.5	50.9	50.8	50.0	0.178	2.92
ALP, U/L	221	206	200	204	1.467	40.3	132	133	146	117	0.401	59.4
ALT, U/L	51.4	46.3	50.6	50.8	0.376	6.8	64.4	62.4	66.1	58.2	1.333	8.57
AST, log	1.62	1.51	1.59	1.55	1.782	0.448	1.83	1.85	1.75	1.81	1.261	0.325
GGT, U/L	39.5	35.0	36.3	33.0	1.408	11.8	39.6	39.5	40.5	39.2	0.059	11.2
LDH, log	2.77	2.72	2.76	2.74	0.546	0.333	2.85	2.88	2.78	2.87	1.944	0.280
Inflammation												
Globulins, g/L	23.0	24.1	23.5	23.6	0.097	3.56	24.0	22.3	23.3	24.1	0.284	3.98
PROTt, g/L	64.6	63.1	63.3	63.0	1.169	3.41	74.5	73.3	74.1	74.0	0.221	3.38
Haptoglobin, log	1.84	1.86	2.00	1.87	0.799	0.456	1.98	2.01	2.03	2.13	0.235	0.578
Oxidative stress metabolites												
ROM, mgH_2_O_2_/100 mL	52.8	49.7	55.1	53.8	0.156	6.02	83.1	82.2	88.9	82.9	0.344	11.6
OXY, μmol HClO/mL	289	279	280	272	0.130	15.5	337	342	339	340	0.837	14.0
Minerals												
Ca, mmol/L	2.68	2.75	2.81	2.84	1.389	0.22	2.75	2.76	2.78	2.77	0.312	0.141
Cl, mmol/L	97.7	98.9	99.2	98.8	1.463	1.68	98.9	98.1	99.8	98.6	0.318	1.76
Fe, μg/dL	117	117	95.0	107	0.280	42.2	131	127	140	143	0.972	29.5
K, mmol/L	5.92	5.83	5.43	5.25	3.505	0.53	7.93	8.16	8.52	8.74	0.866	0.994
Mg, mmol/L	0.910	0.903	0.920	0.886	0.312	0.05	1.10	1.15	1.14	1.17	0.599	0.102
Na, mmol/L	142	142	144	143	1.715	1.68	147	145	147	145	2.177	3.15
P, mmol/L	3.13	2.91	2.91	2.73	0.918	0.32	3.30	3.22	3.30	3.37	0.817	0.288

a CK = creatin kinase; TG = triglycerides; ALP = alkaline phosphatase; ALT = alanine aminotransferase; AST = aspartate aminotransferase; GGT = γ-glutamyl transferase; LDH = lactate dehydrogenase; PROTt = total proteins; ROM = reactive oxygen metabolites; OXY = plasma protection against oxidation (hypochlorite-induced oxidation of protein in plasma).

b CTR = control diet; AP33 = diet with substitution of soybean with 33% of *A. platensis* nucleus; AP66 = diet with substitution of soybean with 66% of *A. platensis* nucleus; AP100 = diet with total substitution of soybean with *A. platensis* nucleus.

c RMSE = root mean square error.

Results of ANOVA for blood sampling period and sex effects and their interaction, and the least square means of blood biochemical parameters for gilts and barrows at T0 and T1 are reported in Table 6. The time of blood collection (T0 and T1) reflects potential differences in the age of pigs at sampling and in dietary treatments. As previously observed, different diets did not affect the blood parameters; nevertheless, the effect of diets has been included in the statistical model; therefore, we can assume that the variation due to the time of blood collection reflects mostly differences in the age of pigs. As evidenced in Table 6, pigs sampled at T0 and T1 showed significant differences for almost all parameters, except for glucose, globulins, Ca, and Cl. In general, when the age effect was significant, mean values of blood parameters were greater in older pigs, with the exception of ALP for liver function indicators.

**Table 6:** Least square means of blood biochemical parameters at the start of the trial, before *A. platensis* administration (T0), and at the end of the trial (T1) for gilts and barrows, and results of ANOVA (F-values and level of significance) for time of blood collection, sex, and their interaction.

Item^a^	T0		T1		F-value^b^			RMSE^c^
	Gilts	Barrows	Gilts	Barrows	Time (T)	Sex (S)	T × S	
Energy-related metabolites								
Glucose, mmol/L	5.61	5.74	5.74	5.88	0.91	0.79	0.06	0.29
Total Cholesterol, mmol/L	2.10	2.31	2.82	2.68	154.67^**^	0.56	15.95^**^	0.92
Creatinine, μmol/L	99.00	90.62	160.72	162.71	853.13^**^	1.14	5.11^*^	14.76
CK, log	3.01	3.03	3.30	3.30	33.22^**^	0.01	0.05	0.31
TG, log	−0.38	−0.34	−0.29	−0.35	4.91^*^	0.57	1.12	0.12
Urea, mmol/L	3.47	4.06	4.26	4.47	26.59^**^	9.97^**^	1.20	0.75
Liver function								
Albumin, g/L	39.27	40.71	50.64	50.49	749.62^**^	1.66	0.82	2.49
ALP, U/L	206.92	207.29	124.37	139.77	135.85^**^	0.76	2.27	41.37
ALT, U/L	50.44	49.14	62.34	62.33	149.58^**^	0.03	1.65	6.85
AST, log	40.59	42.23	71.77	66.06	86.18^**^	0.03	2.40	0.17
GGT, U/L	35.68	36.81	40.02	40.59	33.45^**^	0.12	0.50	4.43
LDH, log	2.75	2.75	2.85	2.85	23.07^**^	0.21	1.58	0.13
Inflammation								
Globulins, g/L	23.55	23.48	23.19	23.57	0.06	0.06	0.80	3.58
PROTt, g/L	62.78	64.20	73.80	74.08	515.38^**^	2.07	0.60	2.97
Haptoglobin, log	1.88	1.91	2.09	1.98	18.86^**^	1.01	5.16^*^	0.21
Oxidative stress metabolites								
ROM, mgH_2_O_2_/100 mL	641.30	680.48	1,017.98	1,089.23	582.88^**^	8.58^**^	0.97	105.26
OXY, μmol HClO/mL	278.61	281.58	338.65	340.09	691.30^**^	0.88	0.12	14.65
Minerals								
Ca, mmol/L	2.75	2.79	2.73	2.80	0.04	3.25	0.23	0.18
Cl, mmol/L	98.90	98.39	98.41	99.25	0.50	0.39	6.63^*^	1.71
Fe, μg/dL	94.17	123.86	139.98	129.92	20.78^**^	2.87	12.19^**^	36.99
K, mmol/L	5.59	5.62	8.14	8.54	457.79^**^	2.91	2.03	0.76
Mg, mmol/L	0.88	0.93	1.12	1.16	446.90^**^	8.05^**^	0.53	0.07
Na, mmol/L	143.20	142.68	145.13	146.72	57.60^**^	1.86	7.13^**^	2.56
P, mmol/L	2.80	3.05	3.29	3.30	57.66^**^	6.65^*^	5.45^*^	0.32

a CK = creatin kinase; TG = triglycerides; ALP = alkaline phosphatase; ALT = alanine aminotransferase; AST = aspartate aminotransferase; GGT = γ-glutamyl transferase; LDH = lactate dehydrogenase; PROTt = total proteins; ROM = reactive oxygen metabolites; OXY = plasma protection against oxidation (hypochlorite-induced oxidation of protein in plasma).

b ^*^: *p* < 0.05; ^**^: *p* < 0.01.

c RMSE = root mean square error.

In contrast, the effect of sex was significant only for urea, ROM, Mg, and P, where gilts showed lower blood concentrations compared to barrows. Finally, a significant interaction was observed between time and sex for cholesterol, creatinine, haptoglobin, Cl, Fe, Na, and P. However, a clear sex-related difference in the pattern of variation was evident only for the blood content of Fe, which sharply increased in gilts but remained nearly unchanged in barrows moving from T0 to T1, and for cholesterol and haptoglobin, which showed a greater increase in gilts than in barrows at increasing the age of sampling.

## Discussion

This study investigated the effects of using AP as a main dietary ingredient on the health status of growing and finishing pigs. Local effects on gut morphology and systemic effects assessed through blood metabolic parameters were evaluated. Outcomes of this study complete the results concerning effects of such a use on growth performance and carcass traits previously reported by Don et al. (29), providing additional insights into the technical feasibility of replacing soybean meal in the diets of growing pigs with more environmentally sustainable ingredients such as this cyanobacterium.

The feeding trial involved growing and finishing pigs slaughtered at heavy body weight, close to 175 kg, and mature age, approximately 9 months, in compliance with the rules governing the production of typical Italian dry-cured hams (34). As fully detailed in Don et al. (29), the substitution of soybean meal with AP, even at full replacement, did not significantly affect growth performance, feed efficiency, and primary carcass traits of pigs.

### Gut morphology

The microscopic structure of the intestine, primarily expressed considering VH, VW, CD, and the ratio VH/CD, is an agreed indicator of the health and functionality of the gut (35). Longer and wider villi are associated with an increased absorptive surface area and subsequent appropriate digestive enzyme action, whereas shallower crypts may indicate greater villus stability and a reduced need for replacement of absorptive epithelial cells and specialized enterocytes continuously renewed from crypt-base stem cells (36, 37). The cells on the tip of the villi are more active in the function of nutrient absorption. As several nutritional factors may affect the intestine’s health (38), changes in villus morphology can indicate potential damage from feed-related disorders. It should also be mentioned that a damaged mucosa may provide a reduced contribution to immune responses, particularly through Peyer’s patches and diffuse gut-associated lymphoid tissue (GALT) (39, 40).

Effects of the inclusion of microalgae in the diet on the digestive tract conditions have been investigated primarily in young pigs, whereas no information is available to our knowledge in finishing pigs. In the present study, the progressive replacement of soybean meal with AP as the main dietary protein source did not significantly affect any of the investigated histomorphological gut parameters or intestine inflammation, suggesting that the use of this cyanobacterium as a dietary ingredient, even at a considerable dosage, was not associated with a deterioration of gut morphology. These outcomes contribute to better explaining the negligible effects on the digestibility of diets that we observed in the same feeding trial (30). Nevertheless, the nominal increase in ileal inflammatory score observed in the AP100 group may indicate an engagement of mucosal immune cells at the highest AP inclusion level, leading to a modest increase in inflammatory cell infiltration. Indeed, the capability of AP of stimulating gut immune system has been demonstrated in diverse mammals (41, 42). On the other hand, the use of AP as a supplement in the diets of weaning and weaned piglets, with BW ranging between 5 and 20 kg, exerted controversial effects on the intestinal mucosa. Indeed, Furbeyre et al. (19) reported that, compared to the control group, weaned piglets receiving diets supplemented with AP had higher VHs in the jejunum but not at the ileum, whereas dietary treatment did not affect crypt depth at the jejunum. Conversely, supplementing AP to diets fed to 5 kg BW piglets around weaning significantly reduced the VH at the ileum, whereas it did not affect mucosa architecture at the jejunum (43).

On the other hand, and consistently with our results, Martins et al. (44) reported no significant effects of using AP as a dietary ingredient at 10% for 4 weeks on VH, VW, and CD in the duodenum, jejunum, and ileum of 30 kg BW piglets.

### Blood biochemical parameters

Blood biochemical parameters may provide useful information when assessing the health status of animals, because they reflect systemic metabolism and can signal potential alterations or damage to specific organs, such as the liver and the kidneys (45, 46). Moreover, monitoring the dynamics of blood metabolite concentration in response to the supply of new feeds may help to better understand the physiological implications of their administration and of the optimal level of dietary inclusion (47).

A majority of scientific literature reports blood parameter values from pigs of lower body weight than ours, generally not exceeding 50 kg. Thus, some discrepancies between our blood biochemical parameter concentrations and those reported in other studies may be expected. Overall, the mean values of most biochemical parameters observed at the T0 sampling period in our study fall within the reference ranges reported by Klem et al. (48), Li et al. (45), and Meissner et al. (49) for healthy pigs weighing between 7 and 50 kg. The only exception was albumin, which exceeded the reference values reported by Klem et al. (48) and Meissner et al. (45). Conversely, at the end of the trial (T1), the average blood creatinine concentration exceeded the reference values reported by Klem et al. (44) and Li et al. (41). Additionally, total protein and K levels exceeded the reference ranges reported by Klem et al. (44) and Meissner et al. (45), while Na levels exceeded those reported by Li et al. (41). AST and LDH exceeded the reference values from Meissner et al. (45) only. On the other hand, our T1 biochemical parameters were largely consistent with those reported by Abeni et al. (50), whose study involved similar genetic lines and production systems, and the BW of pigs at blood collection was close to 100 kg. This finding supports the notion that, as body weight increases, the physiological stress associated with growth and metabolic requirements intensifies, adding greater challenges to hepatic function and the regulation of homeostasis.

Conversely, we did not find established reference thresholds for the oxidative stress category. Hence, we classified these metabolites into tertiles. Regarding ROM, such classification evidenced that 30 animals fell within the third tertile at T0 (> 55.7 mg H_2_O_2_/100 mL) and 28 at T1 (> 87.8 mg H2O2/100 mL), of which 15 pigs were in common between the two sampling times (17.4%). In addition, for 29 and 31 animals we observed low levels of OXY at T0 (< 271.7 μmol HClO/mL) and T1 (< 333 μmol HClO/mL), respectively, as they resided in the first tertile. This test provides a total evaluation of the antioxidant level of endogenous antioxidants, such as albumin, bilirubin, reduced glutathione, and uric acid, and exogenous antioxidants, such as vitamin C/E and polyphenol, while also quantifying antioxidants called shock-adsorbents (e.g., mucopolysaccharides). Of these, 8 pigs (9.3%) showed low levels of OXY at both sampling times.

In the present study, the age at blood sampling significantly influenced most blood biochemical parameters, with a general increase of mean values from T0 to T1, except for a few parameters. Age is a well-established source of variation in blood parameters among growing pigs (51). Concerning energy-related blood metabolites, the results of our study are in good agreement with previous findings that reported age-related increases in cholesterol (48, 52), creatinine (53), cytokinin (52), and urea (53). More controversial is the trend of blood glucose with age. In our study, glucose concentrations remained unchanged between sampling periods, consistent with the findings of Dubreuil and Lapierre (47). In contrast, other studies have reported either an increase (52) or a decrease (53, 54) in glucose concentrations with advancing age. It is worth noting that, beyond age at sampling, blood glucose levels are affected by other factors, including the amount of feed energy provided in the diet, the interval between the last meal and blood collection, and the sampling methodology (45, 47).

The trend of age-related variation in liver function indicators is generally consistent with previous findings. We observed an increase in albumin, as reported by Dubreuil et al. (47) and Hellweing et al. (48), and ALT (52), along with a decrease in ALP (45, 48, 54). Lactate dehydrogenase, an important stress indicator enzyme to monitor preslaughter conditions, was found to be elevated in nearly all animals by the end of the trial (T1). During intense muscular activity or muscle damage, LDH is released into the bloodstream due to muscle cell membrane rupture, making it a marker of significant physical exertion (55). This elevation is typical during the final fattening stage, where physical stress is heightened after handling or transportation (56, 57).

Among inflammatory blood metabolites, the increase in haptoglobin from T0 to T1 fits well with the findings of Pomorska-Mól et al. (58), who reported a strong positive correlation between age and this acute-phase protein concentration. Moreover, Petersen et al. (59) reported that pigs aged 20–25 weeks had higher haptoglobin concentrations than pigs aged 10–14 weeks. Interestingly, the increase in age-related haptoglobin in our study was greater in gilts than in barrows, with a significant interaction between sex and sampling time (*p* < 0.05).

Regarding the dynamics of blood minerals with age, our results evidenced an increasing trend for all minerals except Ca and Cl, whose content did not significantly change between T0 and T1. These findings agree with the results of Dubreuil and Lapierre (51) for most minerals, but not for K, which has been reported unchanged with age in that study.

Regarding oxidative stress, at both sampling times, one-third of the animals exhibited elevated oxidative stress, characterized by high ROM and low levels of antioxidant compounds (OXY), highlighting an inadequate production or renewal of antioxidant molecules and/or their progressive depletion by an increased formation of ROM (60). Interestingly, the same animals did not consistently show these changes at both sampling times, suggesting that these were not chronic or persistent conditions but rather represented an adaptive response to stress conditions without impairment of the response itself. In addition, as also evidenced in our study, oxidative stress has been reported to be closely associated with age, with younger pigs having lower antioxidant defenses and older pigs showing higher oxidative markers. This pattern suggests that stress during rearing tends to increase with age, likely due to the cumulative metabolic load and environmental changes (61, 62).

Sex-related differences had a limited influence on the variation of biochemical blood parameters, and gilts and barrows showed comparable values for most metabolites at both blood sampling periods, with few exceptions. Notably, gilts evidenced a lower serum urea content than barrows at both T0 and T1. This finding contrasts with the outcomes of Olivàn et al. (63), who reported an inverse trend, observing higher blood urea levels in gilts than in barrows slaughtered at an average BW of 111 kg. It is known that blood urea is influenced by the time elapsed after feeding, making it highly variable (64), as also reflected in our samples (CV = 23.7%). Blood urea has been shown to have a linear, negative relationship with feed efficiency and lean gain, particularly during the growing phase at 60–70 kg BW (60). This evidence aligns with the outcomes of our feeding trial, in which gilts displayed greater growth rates, improved gain-to-feed ratios, and lower backfat thickness compared with barrows (29). In recent years, there has been growing interest in improving nitrogen utilization efficiency in order to support a more sustainable pig production. Blood urea concentration is positively correlated with urea nitrogen excretion (65–67). Moreover, while the concentration is affected by nutritional factors, non-nutritional factors, such as digestive and enzymatic efficiency, which are heritable traits, also play a role and have become the focus of selection (68). In this context, diverse studies highlighted that genetics affect blood urea, where genetic lines with higher feed efficiency show lowered blood urea levels (50, 69). Given that breeding selection is always improving, it is necessary to regularly update reference thresholds, also accounting for different genetics, ages, or production systems.

Conversely to blood urea content, we observed a plasma ROM level higher in barrows than in gilts at both T0 and T1 blood sampling periods. These findings agree with what was recently reported by Heras-Molina (70) in a transcriptome study, where they highlighted that male pigs had higher expression of genes related to oxidative metabolism, with a consequent greater production of reactive oxygen species and nitric oxide. This suggests the existence of sex-specific metabolic regulation of oxidative pathways.

We also observed a significant interaction between sex and age at blood sampling for total cholesterol, haptoglobin, and Fe blood concentrations. All of these parameters evidenced a greater increase in gilts than in barrows at an increasing age of sampling, such that gilts showed lower levels at T0 but higher levels at T1 compared with barrows. Moreover, Lee et al. (71) reported greater plasma total cholesterol content in gilts than in barrows sampled at an average BW of 100 kg. For plasma haptoglobin content, the evidence in the literature is controversial: Pineiro et al. (72), in agreement with our results, found greater plasma haptoglobin level in sows than in adult boars, whereas Lipperheide et al. (73) reported that differences between sexes of apparently healthy animals seemed to have no influence on the plasma concentration of haptoglobin.

In the present study, the administration of AP as a feed ingredient of the diet, even at high dosage, appeared metabolically neutral, as feeding strategies did not significantly impact blood biochemical parameters. More generally, throughout the entire feeding trial described by Don et al. (29), the AP dietary treatments did not exert any detrimental effects on animal health, and health-related issues were fully comparable across the different dietary groups.

The initial blood collection (T0) was carried out during the acclimation period, when all pigs were fed the same diet, thereby minimizing potential confounding factors unrelated to dietary differences. Therefore, the absence of a significant effect related to experimental dietary groups at T0 was expected. On the other hand, the second blood sampling (T1) took place after 140 days of the feeding trial, during which soybean meal was partially to fully replaced with AP. The duration and the amount of AP administration allowed for a robust evaluation of its impact on the metabolic profile and overall health status of finishing pigs.

Few studies have investigated the relationship between dietary use of AP and biochemical blood markers in pigs (74, 75). Specifically, to date, no studies have been conducted on finishing pigs accomplishing a complete substitution of protein sources. As a result, the comparison with existing literature is challenging. Neumann et al. (76) emphasized that a crucial aspect of substituting soybean meal with AP at high inclusion levels is the need for adequate amino acids supplementation, balancing functional amino acids. In our study, amino acid levels were well balanced across all four feeding strategies (Table 1), which contributed to modulating response regulation and supporting optimal metabolic functions (77). This evidence is confirmed by the lack of any effect of AP dietary inclusion on blood urea content, a metabolic parameter used as an indicator of the level of amino acid utilization by the animal and of potential amino acid imbalance (47).

In agreement with our results, Nedeva et al. (74) observed that the supplementation of the diet with 2–3 g of AP did not affect liver function in piglets. Similarly, Saeid et al. (28) reported that the supplementation of diets for fattening pigs with *Spirulina Maxima* enriched with Cu for 87 days of feeding trial, from 21 to 106 kg BW, did not affect any biochemical parameter in blood serum, with the only exception of total cholesterol level, which was lower in the AP fed group.

Conversely, when AP was used as a feed ingredient at 10% inclusion level for 4 weeks in the diet fed to pigs from 12 to 30 kg BW, dos Santos Madeira et al. (75) observed an increase in total cholesterol. A consistent pattern of increased total cholesterol, triacylglycerols, and total lipids in broilers fed until 35 days of age with 15% AP in partial replacement of soybean meal has also been reported by Spinola et al. (78) and by Lopes et al. (79), who explained that this lipemic boost effect could be due to an enhancement of fat absorption in the intestinal tract promoted by AP addition. In our study, we observed no significant change in triglycerides and total cholesterol levels in the blood of pigs fed AP.

Moreover, and in contrast with our results, a pejorative effect on hepatic enzymes has been associated with the use of AP as a feed ingredient, both in weaned pigs until 30 kg BW (75), with an increase in plasma levels of ALT and ALP, and in broiler, with an increase in plasma levels of ALP and GGT (22, 79). However, all of these authors have pointed out that the observed variations in hepatic function lacked clinical relevance, as the enzymatic activity levels remained within established reference ranges for both pigs and birds, in agreement with our results.

Regarding oxidative status markers, no differences were observed among the four treatments. In growing pigs, supplementation with 0.1% AP resulted in an increased activity of the antioxidant enzyme glutathione peroxidase (21). Similar effects have been observed with analogous AP supplementation in chicken broilers (80). Although direct comparisons are limited by differences in species, growth periods, and administration protocols, it can be hypothesized that the antioxidant activity of AP is likely more pronounced when directly assessing the enzymatic antioxidant response rather than measuring reactive oxygen species and their by-products (81). In addition, the absence of detectable antioxidant effects of AP in the present study may be associated with the heavy body weight of the pigs, as blood samples were collected close to the slaughter age, whereas antioxidant responses to AP have been more frequently reported in younger animals (21). Finally, the high level of AP administration and the long duration of the feeding trial may have allowed physiological adaptation of the pigs to the diet.

Concerning inflammation biomarkers, globulins, total proteins, and haptoglobin were observed to not affect AP administration, even though haptoglobin showed the nominally highest mean value in the AP100 diet. These findings agree with the results of Furbeyre et al. (19), who reported that blood haptoglobin concentration was not affected by dietary supplementation with AP in the diets of weaned piglets. Beyond its role as an indicator for infections and acute inflammations (82), haptoglobin has also been suggested also as an indicator of non-inflammatory and psychological stress response (83, 84). We can hypothesize that the multifactorial influences reflected by this biomarker during the stressful and delicate phase of the end of finishing may have masked the potential beneficial effects of AP administration. Moreover, the absence of significant anti-inflammatory effects may be partly attributable to the sensitivity of the parameters assessed. While biomarkers such as haptoglobin are indicators of inflammatory status, detecting the immune system modulation often requires the evaluation of more specific molecular markers, such as cytokines (e.g., interleukins, tumor necrosis factor *α*), or tissue-level responses. Therefore, further investigation involving the direct measurement of antioxidant enzyme activities (e.g., catalase and glutathione peroxidase) and as molecular markers of inflammation (e.g., cytokines and transcription factor expression) may provide deeper insights and contribute to addressing this gap in our study.

In conclusion, the absence of adverse effects on gut morphology, intestinal inflammation, and systemic blood biochemical parameters indicates that dietary AP inclusion was nutritionally safe and metabolically neutral under the experimental conditions adopted. These findings align with previous findings in broilers (22, 77) and further support the use of AP as a safe alternative protein source in pig diets, rather than merely a functional additive. This highlights AP as a viable option for the feed industry in the context of sustainability, particularly if production costs can be lowered to ensure the economic affordability of this novel feed source.

## Data Availability

The original contributions presented in the study are included in the article/supplementary material, further inquiries can be directed to the corresponding author.

## References

[1] Parisi G Tulli F Fortina R Marino R Bani P Dalle Zotte A . Protein hunger of the feed sector: the alternatives offered by the plant world. Ital J Anim Sci. (2020) 19:1204–25. doi: 10.1080/1828051X.2020.1827993

[2] Parrini S Aquilani C Pugliese C Bozzi R Sirtori F. Soybean replacement by alternative protein sources in pig nutrition and its effect on meat quality. Animals. (2023) 13:494. doi: 10.3390/ani13030494, 36766383 PMC9913794

[3] Makkar HPS. Review: feed demand landscape and implications of food-not feed strategy for food security and climate change. Animal. (2018) 12:1744–54. doi: 10.1017/S175173111700324X, 29198265

[4] Wilkinson JM Lee MRF. Review: use of human-edible animal feeds by ruminant livestock. Animal. (2018) 12:1735–43. doi: 10.1017/S175173111700218X, 28893333

[5] Mazzocchi C Orsi L Zilia F Costantini M Bacenetti J. Consumer awareness of sustainable supply chains: a choice experiment on Parma ham PDO. Sci Total Environ. (2022) 836:155602. doi: 10.1016/j.scitotenv.2022.155602, 35523351

[6] Holman BWB Malau-Aduli AEO. *Spirulina* as a livestock supplement and animal feed. J Anim Physiol Anim Nutr (Berl). (2013) 97:615–23. doi: 10.1111/j.1439-0396.2012.01328.x, 22860698

[7] Poppi DP McLennan SR. Nutritional research to meet future challenges. Anim Prod Sci. (2010) 50:329–38. doi: 10.1071/AN09230

[8] Taelman SE De Meester S Van Dijk W da Silva V Dewulf J. Environmental sustainability analysis of a protein-rich livestock feed ingredient in the Netherlands: microalgae production versus soybean import. Resour Conserv Recycl. (2015) 101:61–72. doi: 10.1016/j.resconrec.2015.05.013

[9] Altmann BA Rosenau S. Spirulina as animal feed: opportunities and challenges. Foods. (2022) 11:965. doi: 10.3390/foods11070965, 35407052 PMC8997485

[10] Martins CF Ribeiro DM Costa M Coelho D Alfaia CM Lordelo M . Using microalgae as a sustainable feed resource to enhance quality and nutritional value of pork and poultry meat. Foods. (2021) 10:2933. doi: 10.3390/foods10122933, 34945484 PMC8701271

[11] Altmann BA Neumann C Rothstein S Liebert F Mörlein D. Do dietary soy alternatives lead to pork quality improvements or drawbacks? A look into micro-alga and insect protein in swine diets. Meat Sci. (2019) 153:26–34. doi: 10.1016/j.meatsci.2019.03.001, 30861487

[12] Skrede A Mydland LT Ahlstrøm Ø Reitan KI Gislerød HR Øverland M. Evaluation of microalgae as sources of digestible nutrients for monogastric animals. J Anim Feed Sci. (2011) 20:131–142.

[13] Wlaźlak S Biesek J. Spirulina platensis and *Chlorella vulgaris* in poultry nutrition—a review of current research and potential opportunities. Poult Sci. (2025) 104:105456. doi: 10.1016/j.psj.2025.105456, 40570455 PMC12241973

[14] Sweeney T O’Doherty JV. Marine macroalgal extracts to maintain gut homeostasis in the weaning piglet. Domest Anim Endocrinol. (2016) 56 Suppl:S84–9. doi: 10.1016/j.domaniend.2016.02.002, 27345326

[15] Stokes CR. The development and role of microbial-host interactions in gut mucosal immune development. J Anim Sci Biotechnol. (2017) 8:12. doi: 10.1186/s40104-016-0138-0, 28149511 PMC5270223

[16] Alagbe EO Schulze H Adeola O. Dietary Spirulina effects in *Eimeria* -challenged broiler chickens: growth performance, nutrient digestibility, intestinal morphology, serum biomarkers, and gene expression. J Anim Sci. (2024) 102:skae186. doi: 10.1093/jas/skae186, 38995102 PMC11306789

[17] Ansari MS Hajati H Gholizadeh F Soltani N Alavi SM. Effect of different levels of Spirulina platensis on growth performance, intestinal morphology, gut microflora, carcass characteristics and some blood parameters in broiler chickens. J Physiol Res. (2018) 2:186–197.

[18] Grinstead GS Tokach MD Dritz SS Goodband RD Nelssen JL. Effects of *Spirulina platensis* on growth performance of weanling pigs. Anim Feed Sci Technol. (2000) 83:237–47. doi: 10.1016/S0377-8401(99)00130-3 10764072

[19] Furbeyre H van Milgen J Mener T Gloaguen M Labussière E. Effects of dietary supplementation with freshwater microalgae on growth performance, nutrient digestibility and gut health in weaned piglets. Animal. (2017) 11:183–92. doi: 10.1017/S1751731116001543, 27452961

[20] Martins CF Matzapetakis M Ribeiro DD Pinho M Freire JPB Prates JAM . PSVI-18 the use of Spirulina in piglet’s diets: effects on growth performance, selected biochemical blood parameters and small intestine mucosa morphology and metabolomics profiles. J Anim Sci. (2019) 97:469–70. doi: 10.1093/jas/skz258.925

[21] Liu X Han YS Kim IH. Growth performance, nutrient digestibility, antioxidant enzyme activity, and fecal microbial flora in growing pigs fed diets containing Spirulina. Can J Anim Sci. (2019) 99:840–7. doi: 10.1139/cjas-2018-0168

[22] Spínola MP Costa MM Prates JAM. Analysing the impact of Spirulina intake levels on performance parameters, blood health markers and carcass traits of broiler chickens. Animals. (2024) 14:1964. doi: 10.3390/ani14131964, 38998076 PMC11240424

[23] Jégou M Gondret F Vincent A Tréfeu C Gilbert H Louveau I. Whole blood Transcriptomics is relevant to identify molecular changes in response to genetic selection for feed efficiency and nutritional status in the pig. PLoS One. (2016) 11:e0146550. doi: 10.1371/journal.pone.0146550, 26752050 PMC4709134

[24] Anderson NL Anderson NG. The human plasma proteome. Mol Cell Proteomics. (2002) 1:845–67. doi: 10.1074/mcp.R200007-MCP200, 12488461

[25] Ginsburg GS Haga SB. Translating genomic biomarkers into clinically useful diagnostics. Expert Rev Mol Diagn. (2006) 6:179–91. doi: 10.1586/14737159.6.2.179, 16512778

[26] El-Deeb MM Abdel-Gawad M Abdel-Hafez MAM Saba FE Ibrahim EMM. Effect of adding Spirulina platensis algae to small ruminant rations on productive, reproductive traits and some blood components. Acta Sci. (2023) 45:e57546. doi: 10.4025/actascianimsci.v45i1.57546

[27] EL-Sabagh MR Abd Eldaim MA Mahboub DH Abdel-Daim M. Effects of Spirulina platensis algae on growth performance, Antioxidative status and blood metabolites in fattening lambs. J Agric Sci. (2014) 6:92–98. doi: 10.5539/jas.v6n3p92

[28] Saeid A Chojnacka K Korczyński M Korniewicz D Dobrzański Z. Effect on supplementation of Spirulina maxima enriched with cu on production performance, metabolical and physiological parameters in fattening pigs. J Appl Phycol. (2013) 25:1607–17. doi: 10.1007/s10811-013-9984-8, 24027355 PMC3763163

[29] Don G Giannuzzi D Toscano A Schiavon S Gallo L. Growth performance and carcass traits of growing and finishing pigs fed diets with a partial to total replacement of soybean meal with Spirulina powder. J Anim Sci Biotechnol. (2025) 16:77. doi: 10.1186/s40104-025-01197-7, 40450346 PMC12126896

[30] Schiavon S Don G Giannuzzi D Tono V Toscano A Verdiglione R . Effects of a partial to full replacement of soybean meal with *Arthrospira platensis* on apparent nutrient digestibility of diets for growing pigs. Ital J Anim Sci. (2025) 24:1643–54. doi: 10.1080/1828051X.2025.2536039

[31] Velayudhan BT Daniels KM Horrell DP Hill SR McGilliard ML Corl BA . Developmental histology, segmental expression, and nutritional regulation of Somatotropic Axis genes in small intestine of Preweaned dairy heifers. J Dairy Sci. (2008) 91:3343–52. doi: 10.3168/jds.2008-1014, 18765593

[32] Bakare AG Chimonyo M. Relationship between feed characteristics and histomorphometry of small intestines of growing pigs. S Afr J Anim Sci. (2017) 47:7–15. doi: 10.4314/sajas.v47i1.3

[33] Erben U Loddenkemper C Doerfel K Spieckermann S Haller D Heimesaat MM . Original Article A guide to histomorphological evaluation of intestinal inflammation in mouse models. 2014. 4557–4576. Available online at: www.ijcep.com/PMC4152019 25197329

[34] Bosi P Russo V. The production of the heavy pig for high quality processed products. Ital J Anim Sci. (2004) 3:309–21. doi: 10.4081/ijas.2004.309

[35] Wijtten PJA Langhout DJ Verstegen MWA. Small intestine development in chicks after hatch and in pigs around the time of weaning and its relation with nutrition: a review. Acta Agric Scandinavica Section A. (2012) 62:1–12. doi: 10.1080/09064702.2012.676061

[36] Laudadio V Passantino L Perillo A Lopresti G Passantino A Khan RU . Productive performance and histological features of intestinal mucosa of broiler chickens fed different dietary protein levels. Poult Sci. (2012) 91:265–70. doi: 10.3382/ps.2011-01675, 22184453

[37] Miles RD Butcher GD Henry PR Littell RC. Effect of antibiotic growth promoters on broiler performance, intestinal growth parameters, and quantitative morphology. Poult Sci. (2006) 85:476–85. doi: 10.1093/ps/85.3.476, 16553279

[38] Szabó C Kachungwa Lugata J Ortega ADSV. Gut health and influencing factors in pigs. Animals. (2023) 13:1350. doi: 10.3390/ani13081350, 37106913 PMC10135089

[39] Jung C Hugot J-P Barreau F. Peyer’s patches: the immune sensors of the intestine. Int J Inflam. (2010) 2010:1–12. doi: 10.4061/2010/823710, 21188221 PMC3004000

[40] Ginoski V Cortés Sánchez JL Kahlert S Schulze Holthausen J Grześkowiak Ł Zentek J . Goblet cells and mucus composition in jejunum and ileum containing Peyer’s patches and in Colon: a study in pigs. Animals. (2025) 15:2852. doi: 10.3390/ani15192852, 41096447 PMC12524252

[41] Salahuddin M Abdel-Wareth AAA Stamps KG Carr TL Gray CD Aviña AMW . Dietary Spirulina platensis enhances immune responses and modulates gut microbiota and microbial function in laying hens. Poult Sci. (2025) 104:105800. doi: 10.1016/j.psj.2025.105800, 40945335 PMC12683123

[42] Neyrinck A Taminiau B Walgrave H Daube G Cani P Bindels L . Spirulina protects against hepatic inflammation in aging: an effect related to the modulation of the gut microbiota? Nutrients. (2017) 9:633. doi: 10.3390/nu9060633, 28632181 PMC5490612

[43] Furbeyre H van Milgen J Mener T Gloaguen M Labussière E. Effects of oral supplementation with Spirulina and Chlorella on growth and digestive health in piglets around weaning. Animal. (2018) 12:2264–73. doi: 10.1017/S1751731118000125, 29446342

[44] Martins CF Ribeiro DM Matzapetakis M Pinho MA Kuleš J Horvatić A . Effect of dietary Spirulina (Arthrospira platensis) on the intestinal function of post-weaned piglet: an approach combining proteomics, metabolomics, and histological studies. J Proteome. (2022) 269:104726. doi: 10.1016/j.jprot.2022.104726, 36096433

[45] Li R Wang F Zhang Y Li C Xia C Chen H . Comparison of hematologic and biochemical reference values in specific-pathogen-free 1-month-old Yorkshire pigs and Yorkshire-Landrace crossbred pigs. Can J Vet Res. (2019) 83:285–90. 31571729 PMC6753882

[46] Zhang S Yu B Liu Q Zhang Y Zhu M Shi L . Assessment of hematologic and biochemical parameters for healthy commercial pigs in China. Animals. (2022) 12:2464. doi: 10.3390/ani12182464, 36139329 PMC9494985

[47] Regmi N Wang T Crenshaw MA Rude BJ Liao SF. Effects of dietary lysine levels on the concentrations of selected nutrient metabolites in blood plasma of late-stage finishing pigs. J Anim Physiol Anim Nutr (Berl). (2018) 102:403–9. doi: 10.1111/jpn.12714, 28447366

[48] Klem TB Bleken E Morberg H Thoresen SI Framstad T. Hematologic and biochemical reference intervals for Norwegian crossbreed grower pigs. Vet Clin Pathol. (2010) 39:221–6. doi: 10.1111/j.1939-165X.2009.00199.x, 20051064

[49] Meissner F Dinkelaker J Maier A Pooth J-S Costa Galbas M Schön M . Hematologic and biochemical reference values for anesthetized juvenile German crossbred farm pigs. Sci Rep. (2024) 14:26768. doi: 10.1038/s41598-024-78317-2, 39501056 PMC11538553

[50] Abeni F Petrera F Dal Prà A Rapetti L Crovetto GM Galassi G. Blood parameters in fattening pigs from two genetic types fed diet with three different protein concentrations1. Transl Anim Sci. (2018) 2:372–82. doi: 10.1093/tas/txy069, 32704720 PMC7200405

[51] Dubreuil P Lapierre H. Biochemistry reference values for Quebec lactating dairy cows, nursing sows, growing pigs and calves. Can J Vet Res. (1997) 61:235–239. 9243006 PMC1189410

[52] Hellwing ALF Tauson AH Skrede A. Blood parameters in growing pigs fed increasing levels of bacterial protein meal. Acta Vet Scand. (2007) 49:33. doi: 10.1186/1751-0147-49-33, 17996082 PMC2211288

[53] Lee S Lee SC Jeon Y. Analysis of blood composition by porcine breeding cycle. Vet Med Sci. (2024) 10:e31376. doi: 10.1002/vms3.1376, 38358072 PMC10868141

[54] Falkowski J Milewska W Glogowski J Karpiesiuk K Trzody KH Wydział C . Polish journal of natural sciences body weight, selected blood parameters and semen quality in two age groups of polish LANDRACE artificial insemination boars masa ciała, wybrane wskaźniki krwi i jakość nasienia knurów inseminacyjnych rasy polskiej białej zwislouchej. Polish J Nat Sci. (2014) 29:201–9.

[55] Correa JA Gonyou HW Torrey S Widowski T Bergeron R Crowe TG . Welfare and carcass and meat quality of pigs being transported for two hours using two vehicle types during two seasons of the year. Can J Anim Sci. (2013) 93:43–55. doi: 10.4141/cjas2012-088

[56] Brandt P Aaslyng MD. Welfare measurements of finishing pigs on the day of slaughter: a review. Meat Sci. (2015) 103:13–23. doi: 10.1016/j.meatsci.2014.12.004, 25588903

[57] Ogawa NN Silva GL Barbon APA da C Flaiban KKM da C Silva CA da Rocha LM . Animal welfare assessment and meat quality through assessment of stress biomarkers in fattening pigs with and without visible damage during slaughter Animals 2024 14:700 doi: 10.3390/ani14050700 38473085 PMC10931360

[58] Pomorska-Mól M Kwit K Markowska-Daniel I. Major acute phase proteins in pig serum from birth to slaughter. Bull Vet Inst Pulawy. (2012) 56:553–7. doi: 10.2478/v10213-012-0097-y

[59] Petersen HH Ersbøll AK Jensen CS Nielsen JP. Serum-haptoglobin concentration in Danish slaughter pigs of different health status. Prev Vet Med. (2002) 54:325–35. doi: 10.1016/S0167-5877(02)00054-5, 12163249

[60] Brambilla G Civitareale C Ballerini A Fiori M Amadori M Archetti LI . Response to oxidative stress as a welfare parameter in swine. Redox Rep. (2002) 7:159–63. doi: 10.1179/135100002125000406, 12189046

[61] Buchet A Belloc C Leblanc-Maridor M Merlot E. Effects of age and weaning conditions on blood indicators of oxidative status in pigs. PLoS One. (2017) 12:e0178487. doi: 10.1371/journal.pone.0178487, 28542567 PMC5443573

[62] Guevara RD Pastor JJ López-Vergé S Manteca X Tedo G Llonch P. Physiology, gene expression, and behavior as potential indicators of oxidative stress in piglets. BMC Vet Res. (2024) 20:471. doi: 10.1186/s12917-024-04320-4, 39415196 PMC11481391

[63] Oliván M González J Bassols A Díaz F Carreras R Mainau E . Effect of sex and RYR1 gene mutation on the muscle proteomic profile and main physiological biomarkers in pigs at slaughter. Meat Sci. (2018) 141:81–90. doi: 10.1016/j.meatsci.2018.03.018, 29621665

[64] Whang KY Easter RA. Blood urea nitrogen as an index of feed efficiency and lean growth potential in growing-finishing swine. Asian Australas J Anim Sci. (2000) 13:811–6. doi: 10.5713/ajas.2000.811

[65] Zervas S Zijlstra RT. Effects of dietary protein and oathull fiber on nitrogen excretion patterns and postprandial plasma urea profiles in grower pigs1,2. J Anim Sci. (2002) 80:3238–46. doi: 10.2527/2002.80123238x, 12542165

[66] Kohn RA Dinneen MM Russek-Cohen E. Using blood urea nitrogen to predict nitrogen excretion and efficiency of nitrogen utilization in cattle, sheep, goats, horses, pigs, and rats1. J Anim Sci. (2005) 83:879–89. doi: 10.2527/2005.834879x, 15753344

[67] Berghaus D Haese E Weishaar R Sarpong N Kurz A Seifert J . Nitrogen and lysine utilization efficiencies, protein turnover, and blood urea concentrations in crossbred grower pigs at marginal dietary lysine concentration. J Anim Sci. (2023) 101:skad335. doi: 10.1093/jas/skad335, 37773762 PMC10583982

[68] Kasper C. Animal board invited review: heritability of nitrogen use efficiency in fattening pigs: current state and possible directions animal 2024 18 101225 doi: 10.1016/j.animal.2024.101225 39013333

[69] Madeira MS Pires VMR Alfaia CM Lopes PA Martins SV Pinto RMA . Restriction of dietary protein does not promote hepatic lipogenesis in lean or fatty pigs. Br J Nutr. (2016) 115:1339–51. doi: 10.1017/S0007114516000453, 26927728

[70] Heras-Molina A Núñez Y Benítez R Pesántez-Pacheco JL García-Contreras C Vázquez-Gómez M . Hypothalamic transcriptome analysis reveals male-specific differences in molecular pathways related to oxidative phosphorylation between Iberian pig genotypes. PLoS One. (2022) 17:e0272775. doi: 10.1371/journal.pone.0272775, 35972914 PMC9380940

[71] Lee C-E Kim N-Y Kim K-I. Effects of gender and gonadectomy on growth and plasma cholesterol levels in pigs. Nutr Res Pract. (2009) 3:38–42. doi: 10.4162/nrp.2009.3.1.38, 20016700 PMC2788163

[72] Piñeiro C Piñeiro M Morales J Andrés M Lorenzo E, Pozo M del, Alava MA Lampreave F. Pig-MAP and haptoglobin concentration reference values in swine from commercial farms. Vet J (2009) 179:78–84. doi:10.1016/j.tvjl.2007.08.010, 17911038

[73] Lipperheide C Diepers N Lampreave F Alava M Petersen B. Nephelometric determination of Haptoglobin plasma concentrations in fattening pigs. J Veterinary Med Ser A. (1998) 45:543–50. doi: 10.1111/j.1439-0442.1998.tb00858.x, 9857832

[74] Nedeva R Jordanova G Kistanova E Shumkov K Georgiev B Abadgieva D . Effect of the addition of *spirulina platensis* on the productivity and some blood parameters on growing pigs. Bulg J Agric Sci. (2014) 20:680–4.

[75] dos Santos Madeira MSM Lopes PAAB Martins CF Assunção JMP Alfaia CMRPM Pinto RMA . Dietary Arthrospira platensis improves systemic antioxidant potential and changes plasma lipids without affecting related hepatic metabolic pathways in post-weaned piglets. BMC Vet Res. (2021) 17:158. doi: 10.1186/s12917-021-02869-y, 33849543 PMC8045302

[76] Neumann C Velten S Liebert F. Improving the dietary protein quality by amino acid fortification with a high inclusion level of micro algae (*Spirulina platensis*) or insect meal (*Hermetia illucens*) in meat type chicken diets. Open J Anim Sci. (2018) 8:12–26. doi: 10.4236/ojas.2018.81002 PMC6210745 30282918

[77] Le Floc’h N Wessels A Corrent E Wu G Bosi P. The relevance of functional amino acids to support the health of growing pigs. Anim Feed Sci Technol. (2018) 245:104–16. doi: 10.1016/j.anifeedsci.2018.09.007

[78] Spínola MP Alfaia CM Costa MM Pinto RMA Lopes PA Pestana JM . Impact of high Spirulina diet, extruded or supplemented with enzymes, on blood cells, systemic metabolites, and hepatic lipid and mineral profiles of broiler chickens. Front Vet Sci. (2024) 11:1342310. doi: 10.3389/fvets.2024.1342310, 38596464 PMC11002084

[79] Lopes PA Alfaia CM Spínola MP Pinto RMA Pestana JM Costa MM . The combined effects of extended feeding with a high level of Arthrospira platensis and a commercial enzyme mix or porcine pancreatin on broilers’ blood cells, plasma metabolites and liver lipid profile. BMC Vet Res. (2025) 21:472. doi: 10.1186/s12917-025-04532-2, 40670977 PMC12269105

[80] Park JH Lee SI Kim IH. Effect of dietary Spirulina (Arthrospira) platensis on the growth performance, antioxidant enzyme activity, nutrient digestibility, cecal microflora, excreta noxious gas emission, and breast meat quality of broiler chickens. Poult Sci. (2018) 97:2451–9. doi: 10.3382/ps/pey093, 29672750

[81] Wu Q Liu L Miron A Klímová B Wan D Kuča K. The antioxidant, immunomodulatory, and anti-inflammatory activities of Spirulina: an overview. Arch Toxicol. (2016) 90:1817–40. doi: 10.1007/s00204-016-1744-5, 27259333

[82] Le Floc’h N Jondreville C Matte JJ Seve B. Importance of sanitary environment for growth performance and plasma nutrient homeostasis during the post-weaning period in piglets. Arch Anim Nutr. (2006) 60:23–34. doi: 10.1080/17450390500467810 16529155

[83] Murata H. Stress and acute phase protein response: an inconspicuous but essential linkage. Vet J. (2007) 173:473–4. doi: 10.1016/j.tvjl.2006.05.008, 16807009

[84] Hennig-Pauka I Menzel A Boehme TR Schierbaum H Ganter M Schulz J. Haptoglobin and C-reactive protein—non-specific markers for nursery conditions in swine. Front Vet Sci. (2019) 6:92. doi: 10.3389/fvets.2019.00092, 31001544 PMC6455069

